# Correction: Heat shock factor-1 alleviates ER-stress in *Caenorhabditis elegans*

**DOI:** 10.1038/s41598-026-50440-2

**Published:** 2026-06-09

**Authors:** Saqib Ahmed, Dániel Kovács, Márton Kovács, Mónika Kosztelnik, Bernadette Hotzi, Tímea Sigmond, Éva Saskői, Viktor Vázsony Vincze, Viktor Erdélyi, Veronika Deák, Ibolya Stiller, Tibor Vellai, János Barna

**Affiliations:** 1 Department of Genetics, Institute of Biology, Eötvös Loránd University, Budapest, Hungary; 2 Institute of Translational Medicine, Semmelweis University, Budapest, Hungary; 3 HUN-REN-SU Cerebrovascular and Neurocognitive Disease Research Group, Budapest, Hungary; 4 Department of Applied Biotechnology and Food Science, Laboratory of Biochemistry and Molecular Biology, University of Technology, Budapest, Hungary; 5 Department of Molecular Biology, Institute of Biochemistry and Molecular Biology, Semmelweis University, Budapest, Hungary; 6 HUN-REN-ELTE Genetics Research Group, Eötvös Loránd University, Budapest, Hungary; 7 Food and Wine Research Institute, Eszterházy Károly Catholic University, Eger, Hungary

Correction to: *Scientific Reports* 10.1038/s41598-026-43060-3, published online 25 March 2026.

In the original version of this Article, Figs. 1 did not display correctly. The original Fig. 1 and the accompanying legend appear below.

**Fig. 1:**
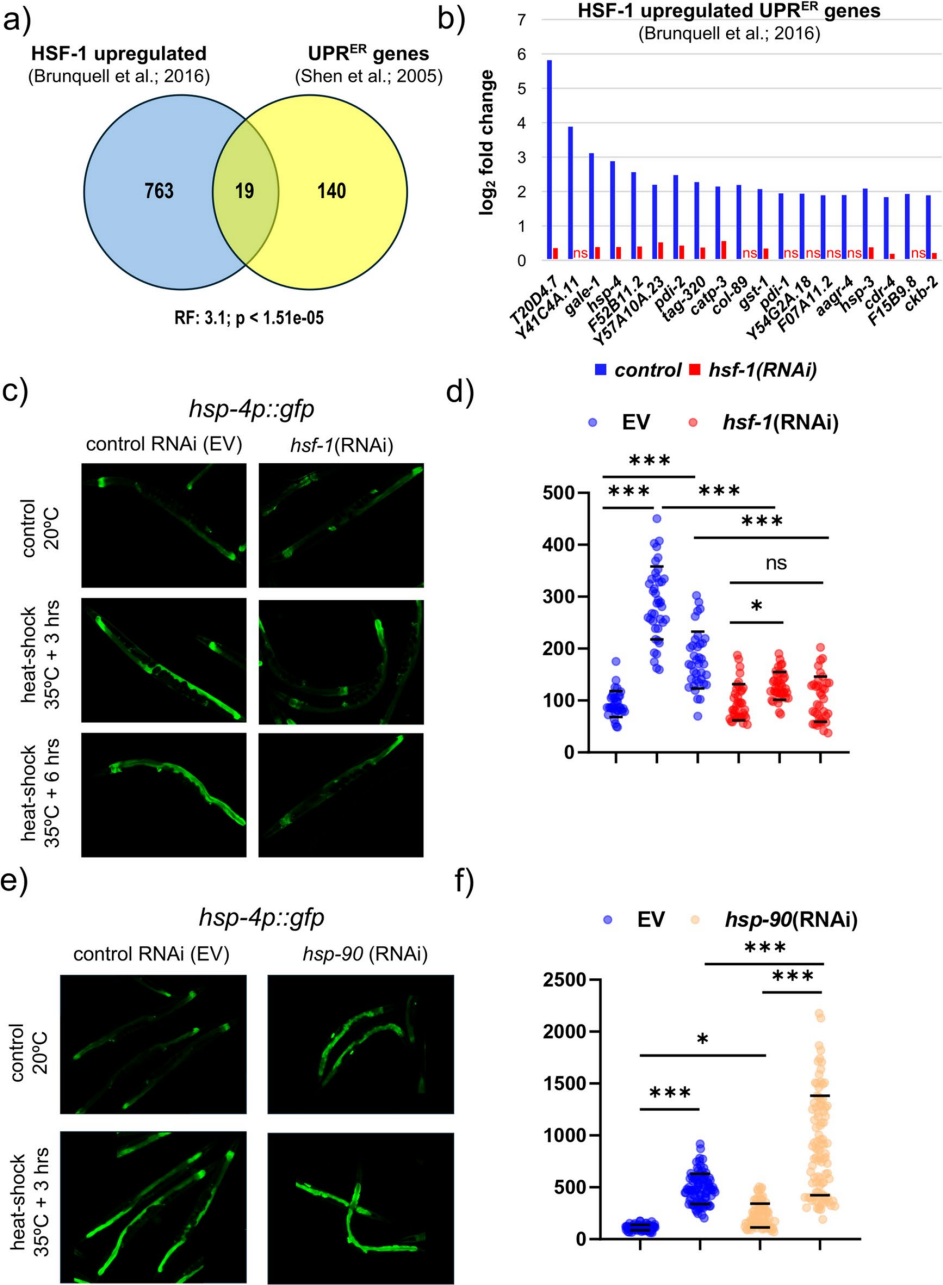
HSF-1 is required for the induction of UPR^ER^ genes upon heat shock. (**a**) Several genes upregulated by HSF-1 following heat stress are also upregulated upon tunicamycin induced ER stress (RF: representation factor). (**b**) Diagram showing the relative levels of UPR^ER^ genes upregulated by HSF-1 following heat stress (ns: not significant). (**c**) Representative fluorescent images showing that the expression of UPR^ER^ reporter *hsp-4p::gfp* is induced by HSF-1 upon heat shock. (**d**) Quantification of the *hsp-4p::gfp* reporter expression in wild-type and *hsf-1(RNAi)* animals under normal conditions and upon heat shock (35 °C). Three replicates of at least 30 animals per strain/trial were analyzed. Data points represent the fluorescent intensity of individual animals, p values were determined using two-way ANOVA with Tukey’s multiple comparisons test; * = *p* < 0.05, ** = *p* < 0.01, *** = *p* < 0.001; error bars represent ± SD.) **e**) Representative fluorescent images showing that the silencing of *hsp-90* induces the expression of UPR^ER^ reporter *hsp-4p::gfp*. (**f**) Quantification of the *hsp-4p::gfp* reporter expression in wild-type and *hsp-90*(RNAi) animals under normal conditions and upon heat shock (35 °C). Three replicates of at least 30 animals per strain/trial were analyzed. Data points represent the fluorescent intensity of individual animals, p values were determined using two-way ANOVA with Tukey’s multiple comparisons test; * = *p* < 0.05 , ** = *p* < 0.01, *** = *p* < 0.001; error bars represent ± SD.) Source data supporting panels a and b are provided in Supplementary Table S1.

In addition, the labels to the accompanying Supplementary Information files were incorrect.

The original Article has been corrected.

## Supplementary Information

Below is the link to the electronic supplementary material.

Supplementary Material 1

Supplementary Material 2

Supplementary Material 3

Supplementary Material 4

Supplementary Material 5

Supplementary Material 6

