# Supplementary material for: Correction: Heat shock factor-1 alleviates ER-stress in Caenorhabditis elegans
Source: Sci Rep. 2026 Jun 9;16:17863. doi: 10.1038/s41598-026-50440-2 (PMC13250110; doi:10.1038/s41598-026-50440-2)
Supplement: Supplementary file 6 — Supplementary Material 6 [file 41598_2026_50440_MOESM6_ESM.pdf]

Heat shock factor-1 alleviates ER-stress in *Caenorhabditis elegans*

Saqib Ahmed[1#], Dániel Kovács[1#], Márton Kovács[1], Mónika Kosztelnik[1,2,3], Bernadette Hotzi[1], Tímea Sigmond[1], Éva Saskői[1], Viktor Vázsony Vincze[1], Viktor Erdélyi[4], Veronika Deák[4], Ibolya Stiller[5], Tibor Vellai[1,6,*], János Barna[1,6,7,*]

[1] Department of Genetics, Institute of Biology, Eötvös Loránd University, Budapest, Hungary

[2] Institute of Translational Medicine, Semmelweis University, Budapest, Hungary

[3] HUN-REN-SU Cerebrovascular and Neurocognitive Disease Research Group, Budapest, Hungary

[4] Department of Applied Biotechnology and Food Science, Laboratory of Biochemistry and Molecular Biology, University of Technology, Budapest, Hungary

[5] Department of Molecular Biology, Institute of Biochemistry and Molecular Biology, Semmelweis University, Budapest, Hungary

[6] HUN-REN-ELTE Genetics Research Group, Eötvös Loránd University, Budapest, Hungary

[7] Food and Wine Research Institute, Eszterházy Károly Catholic University, Eger, Hungary

#: Saqib Ahmed and Dániel Kovács contributed equally to this work.

*: Corresponding authors:

Tibor Vellai: vellai.tibor@ttk.elte.hu, orcid.org/0000-0002-3520-2572

Janos Barna: barna.janos@ttk.elte.hu, orcid.org/0000-0002-9242-0939

Supplementary figures with legends

Fig. S1

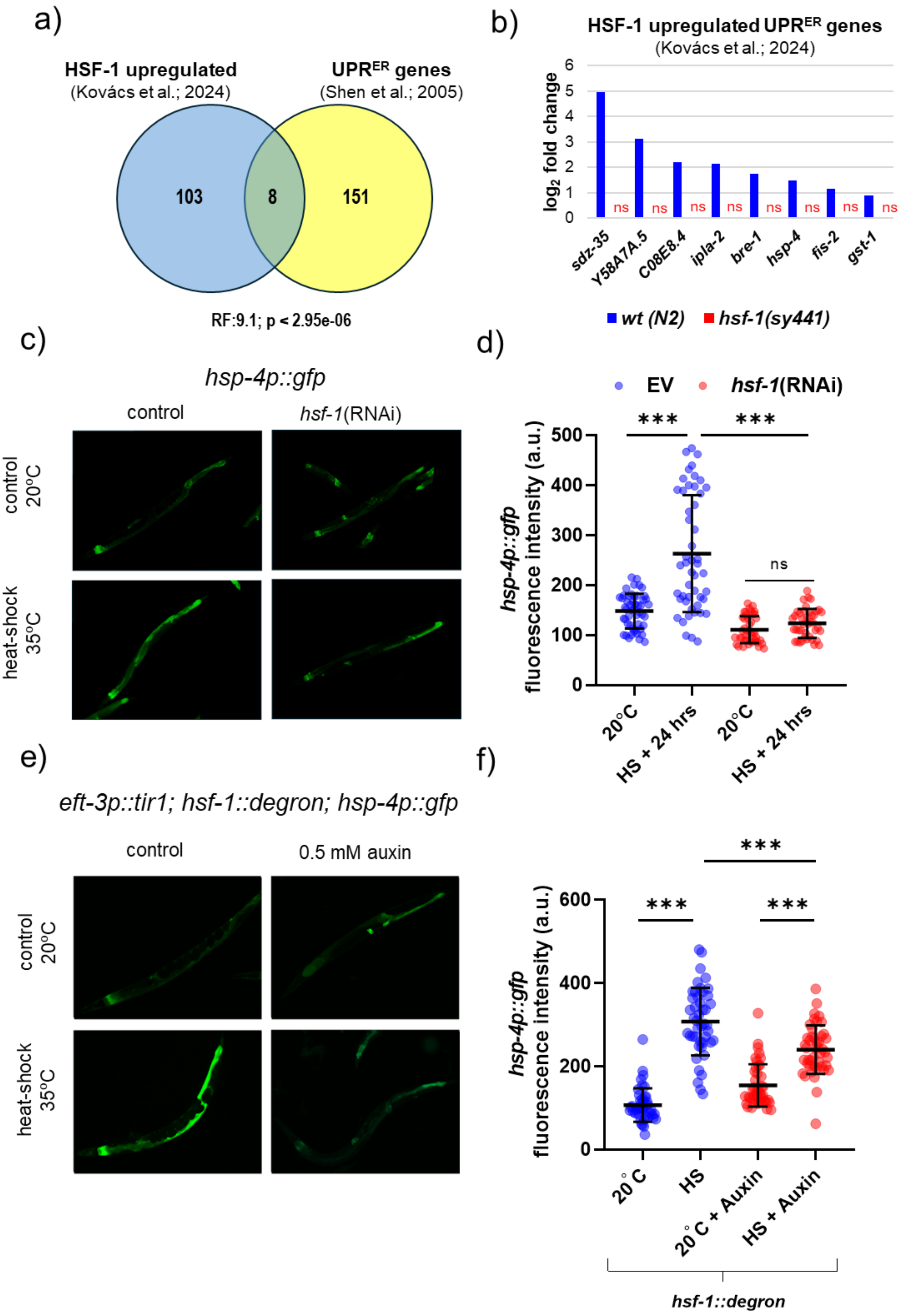

Fig. S1: a) Several genes upregulated by HSF-1 following heat stress are also upregulated upon tunicamycin induced ER stress (RF: representation factor). **b)** Diagram showing the relative levels of UPR^{ER} genes upregulated by HSF-1 following heat stress (ns: not significant). **c)** Representative images showing that the silencing of *hsf-1* significantly attenuated the activation of the reporter *hsp-4p::gfp* upon heat shock (35°C for one hour). **d)** Quantification of the *hsp-4p::gfp* reporter expression in control animals and after silencing *hsf-1* using RNA interference, at normal conditions and upon heat shock. Three replicates of at least 30 animals per strain / trial were analyzed. Data points represent the fluorescent intensity of individual animals, p values were determined using two-way ANOVA with Tukey's multiple comparisons test; *** = $p < 0.001$; error bars represent ± SD. **e)** Representative fluorescent images showing that the induction of UPR^{ER} reporter *hsp-4p::gfp* upon heat stress is attenuated when HSF-1 is depleted using auxin induced degradation. **f)** Quantification of the *hsp-4p::gfp* reporter expression in control animals and after auxin induced depletion of HSF-1 at normal conditions and upon heat shock. Three replicates of at least 30 animals per strain / trial were analyzed. Data points represent the fluorescent intensity of individual animals, p values were determined using one-way ANOVA with Tukey's multiple comparisons test; *** = $p < 0.001$; error bars represent ± SD. Source data underlying panel **a** is provided in Supplementary Tab. S2. Source data underlying panel **d** and **f** is provided in Supplementary Table S2.

Fig. S2

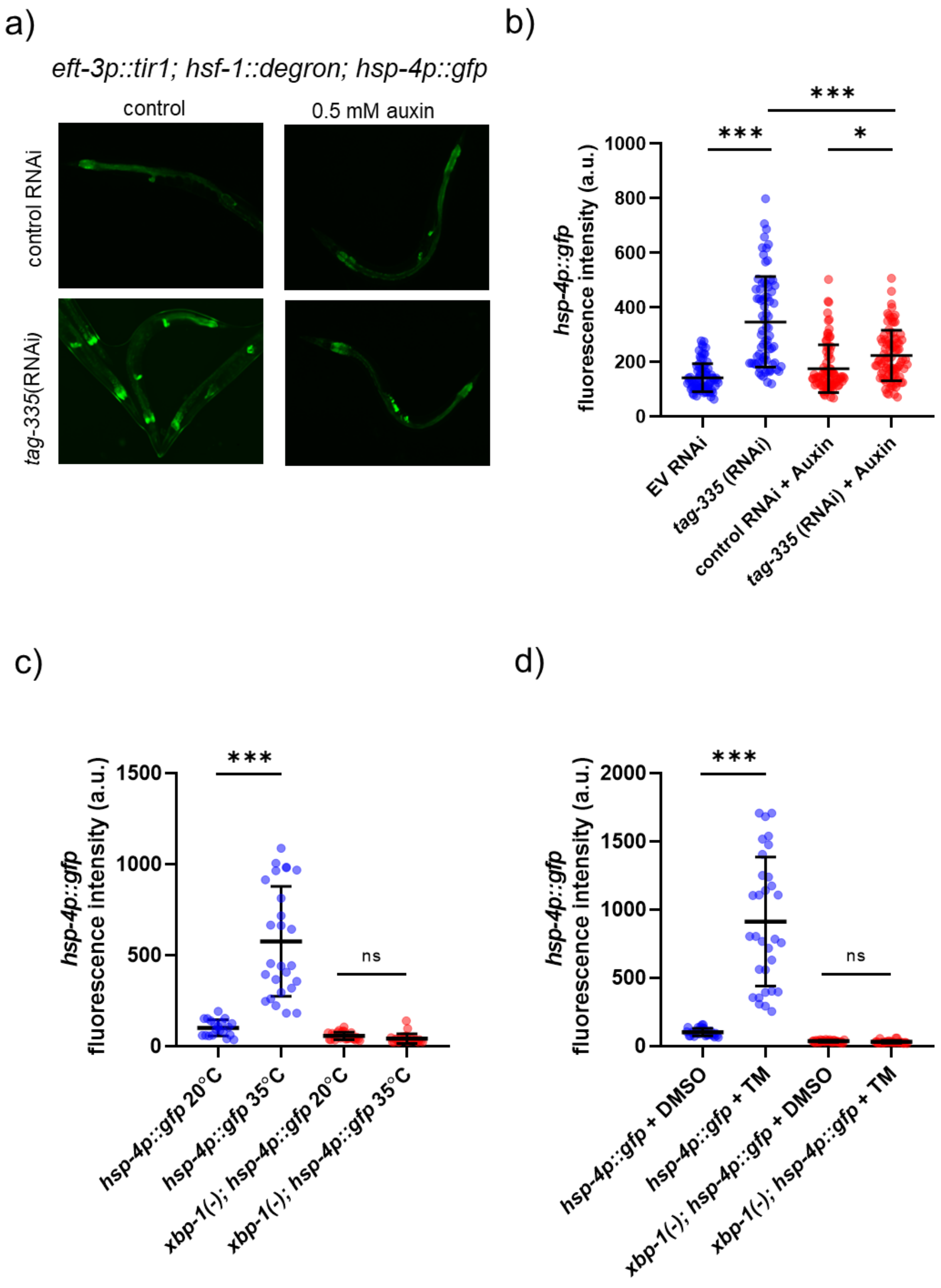
a)
eft-3p::tir1; hsf-1::degron; hsp-4p::gfp
control
0.5 mM auxin
control RNAi
tag-335(RNAi)
b)
hsp-4p::gfp fluorescence intensity (a.u.)
1000
800
600
400
200
0
***
***
*
EV RNAi
tag-335 (RNAi)
control RNAi + Auxin
tag-335 (RNAi) + Auxin
c)
hsp-4p::gfp fluorescence intensity (a.u.)
1500
1000
500
0
***
ns
hsp-4p::gfp 20°C
hsp-4p::gfp 35°C
xbp-1(-); hsp-4p::gfp 20°C
xbp-1(-); hsp-4p::gfp 35°C
d)
hsp-4p::gfp fluorescence intensity (a.u.)
2000
1500
1000
500
0
***
ns
hsp-4p::gfp + DMSO
hsp-4p::gfp + TM
xbp-1(-); hsp-4p::gfp + DMSO
xbp-1(-); hsp-4p::gfp + TM

Fig. S2: a) Auxin induced degradation of HSF-1 reduces *hsp-4* induction under ER stress. Representative fluorescence images of *hsp-4p::gfp* expression in control (EtOH) and auxin treated animals under control conditions or when ER stress was induced using *tag-335*(RNAi). **b)** Quantification of the *hsp-4p::gfp* reporter expression in control and *hsf-1(RNAi)* animals under normal conditions and upon tunicamycin induced ER stress. **c)**, and **c)** Quantification of the *hsp-4p::gfp* reporter expression in wild type (N2) and *xbp-1*(zc12) mutant background under normal conditions and upon heat shock **c**), or tunicamycin induced ER stress **d**). Two replicates of at least 10 animals per strain / trial were analyzed. Data points represent the fluorescent intensity of individual animals, p values were determined using two-way ANOVA with Tukey's multiple comparisons test; * = $p < 0.05$, ** = $p < 0.01$, *** = $p < 0.001$; error bars represent ± SD). Source data is provided in Supplementary Table S2.

Fig. S3

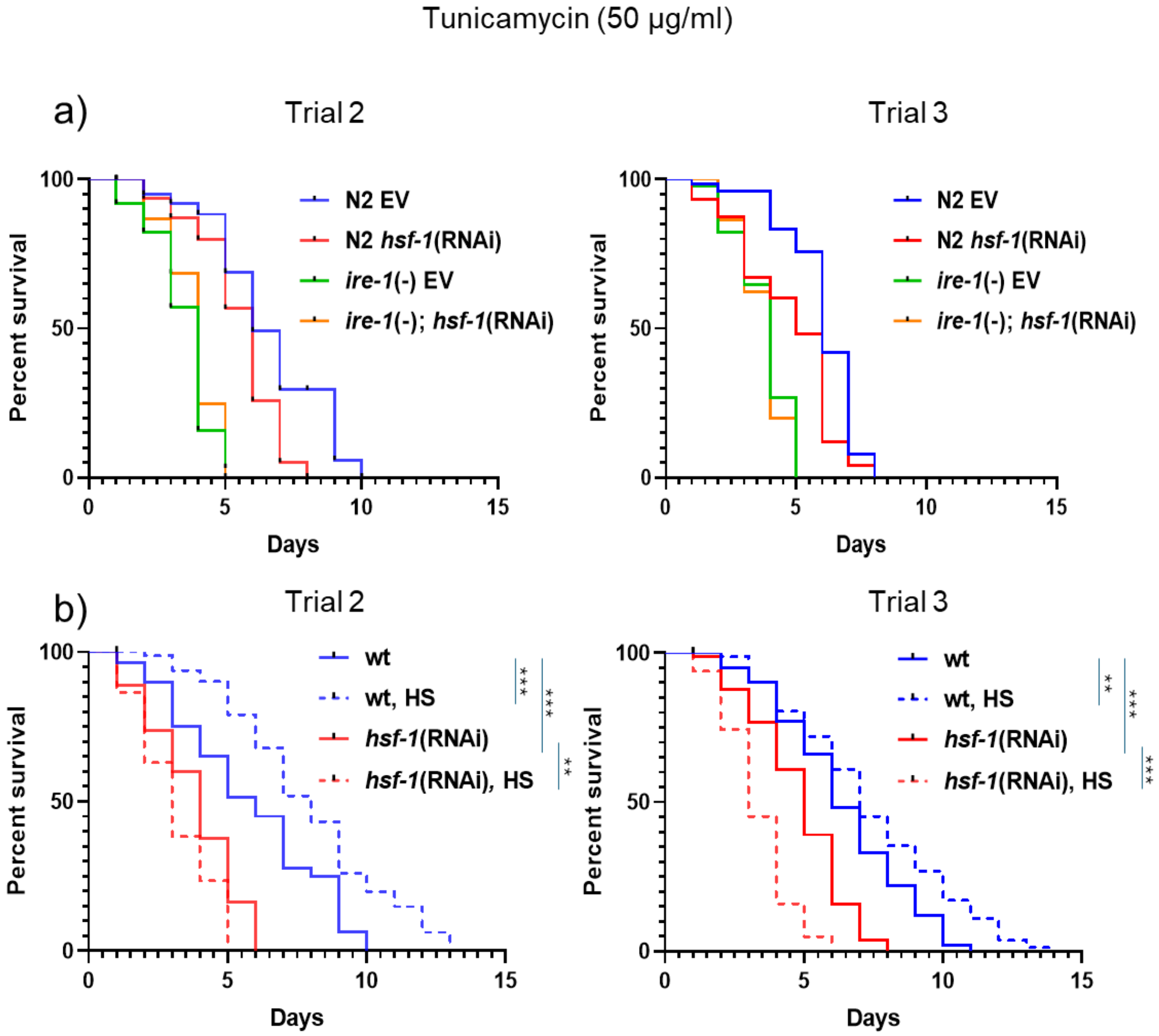

Fig. S3: a) Survival curves of wild-type, *hsf-1(sy441)*, *ire-1(ok799)*, and double mutant *ire-1(ok799); hsf-1(sy441)* animals treated with tunicamycin (50 µg/ml). **b)** Survival curves of wild-type (N2) animals subjected to hormetic heat shock or left untreated, with or without *hsf-1* knockdown by RNAi. For all experiments, three independent replicates with at least 30 animals per strain/condition were analyzed. Statistical comparisons of survival curves were performed using the Log-rank (Mantel–Cox) test (** = $p < 0.05$, ** = $p < 0.01$, *** = $p <$ 0,001*). Source data is provided in Supplementary Table S3.

Fig. S4

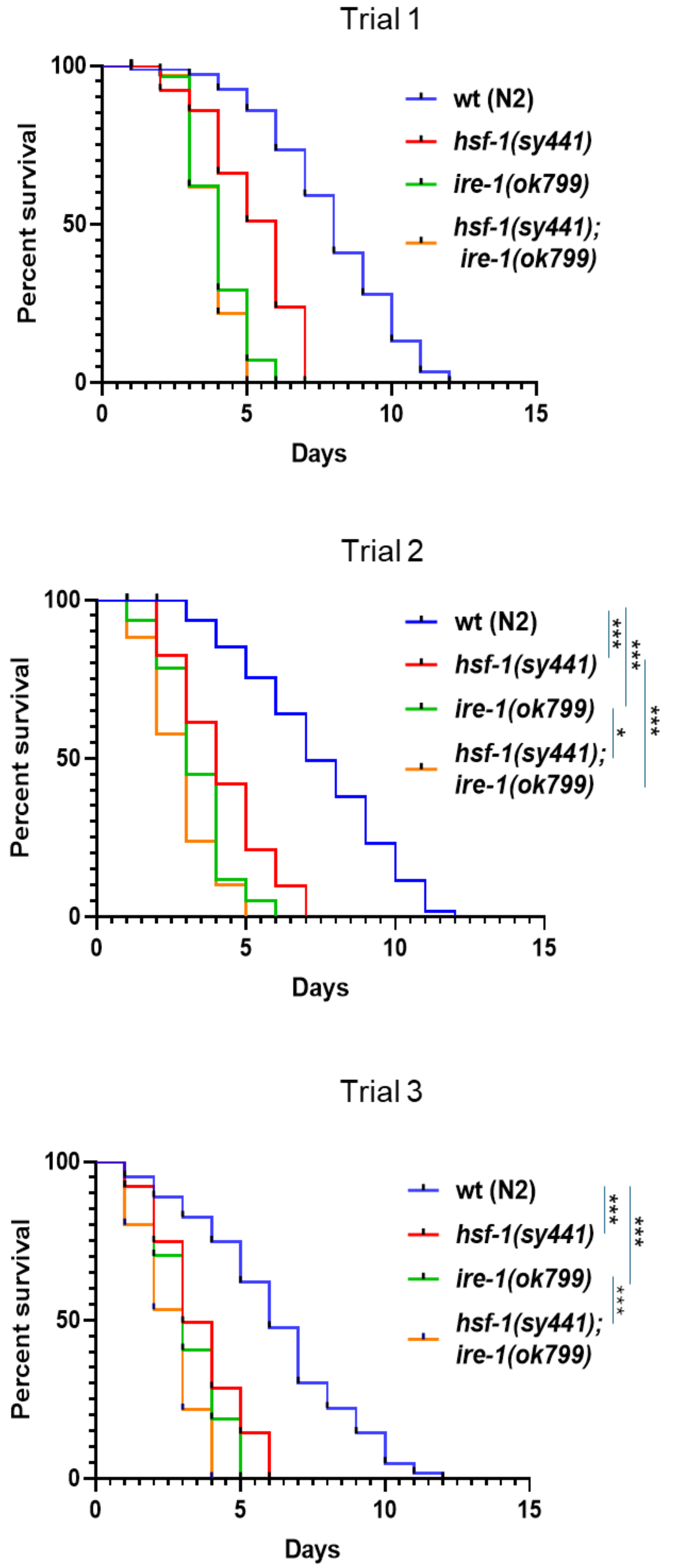
Trial 1
Percent survival
Days
0
50
100
0
5
10
15
wt (N2)
hsf-1(sy441)
ire-1(ok799)
hsf-1(sy441);
ire-1(ok799)
Trial 2
***
***
*
***
Trial 3
***
***
***

Fig. S4: *hsf-1(sy441)* mutants have reduced tolerance to tunicamycin (TM) compared to wild-type animals. We found that the difference in survival between tunicamycin-treated *ire-1(ok799)* single mutants and *ire-1(ok799); hsf-1(sy441)* double mutants was not consistent. For all experiments, three independent replicates with at least 30 animals per strain/condition were analyzed. Statistical comparisons of survival curves were performed using the Log-rank (Mantel–Cox) test (* = $p < 0.05$, ** = $p < 0.01$, *** = $p < 0,001$). Source data is provided in Supplementary Table S3.

Fig. S5

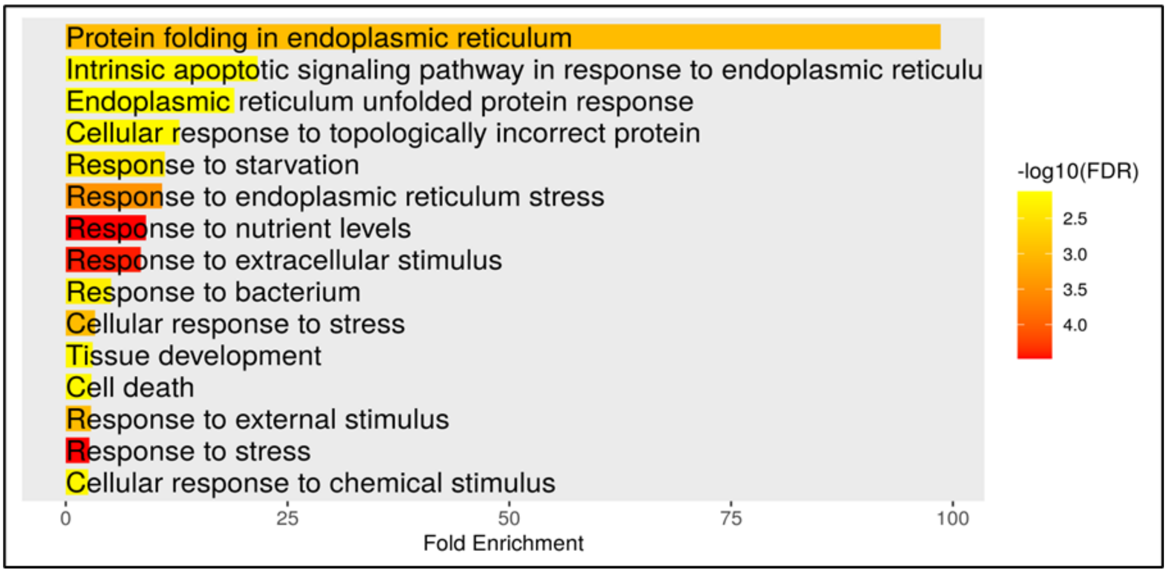

Fig. S5: Gene Ontology (GO) analysis of genes activated by HSF1 and induced upon tunicamycin treatment reveals strong overrepresentation of ER protein folding genes. The horizontal axis indicates fold enrichment, while colors denote significance as –log10(FDR).

Fig. S6

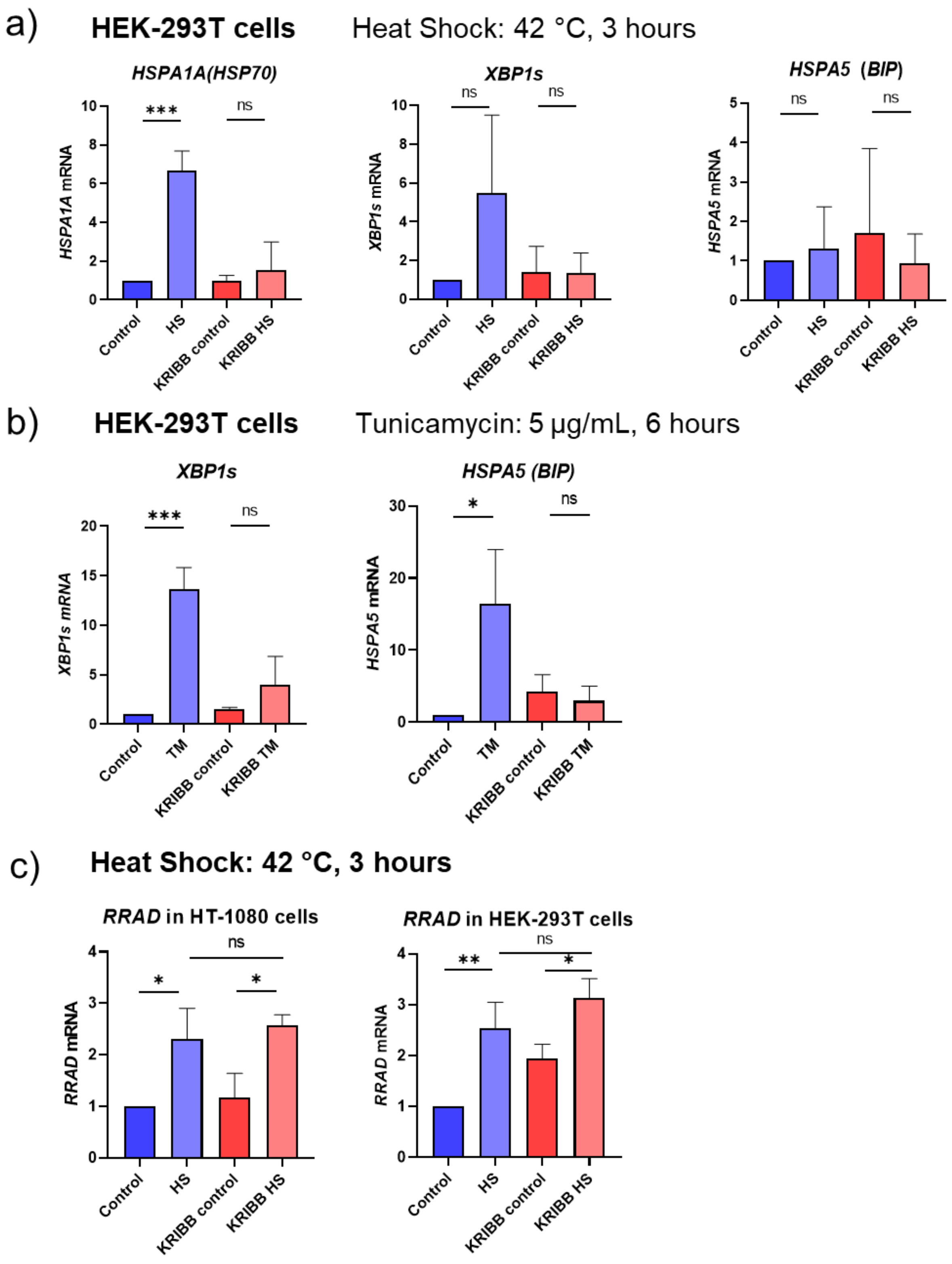

Fig. S6: a) Quantitative RT-PCR to measure mRNA levels of *HSPA1A (HSP70),* spliced *XBP1* (*XBP1s*) and *HSPA5* (*BiP*) following heat shock at 42°C for 3 hours in HEK-293T human cell line, with and without treatment with the HSF1 inhibitor KRIBB11. The results show that

HSP70 mRNA levels increased significantly upon heat shock, but the increase in XBP1s and *HSPA5* levels was not statistically significant. **b)** Quantitative RT-PCR to measure mRNA levels of spliced *XBP1* (*XBP1s*) and *HSPA5* (*BiP*) following tunicamycin treatment (5 μg/mL, for 6 hours) in HEK-293T human cell line, with and without treatment with KRIBB11. Tunicamycin treatment significantly increased the expression levels of both genes in control conditions, but the induction of both genes was suppressed when treated with KRIBB11. **c)** Quantitative RT-PCR to measure mRNA levels of *RRAD* following heat shock at 42°C for 3 hours in HT-1080 and HEK-293T human cell lines, with and without treatment with KRIBB11. *ERAD* was upregulated upon heat shock in a HSF1 independent manner. Error bars indicate SD. p values were determined by 2-way ANOVA; * = $p < 0.05$, ** = $p < 0.01$, *** = $p < 0.001$, ns = not significant (SD = standard deviation; ns = not significant). Source data is provided in Supplementary Table S4.

Supplementary table legends

Table S1: Source data underlying Fig. 1 a, b; Fig. S1 a, b

The table contains the following data on tabs: 1) List of genes that are upregulated by exposure to tunicamycin and dependent on *ire-1*, *xbp-1*, *pek-1* or *atf-6*[1] 2) List of genes that are upregulated by heat shock and dependent on *hsf-1* [2] (Not activated in *hsf-1*(RNAi) animals upon heat shock). 3) Genes that are upregulated by heat shock and dependent on *hsf-1*[3] (Not activated in *hsf-1*(sy441) mutant animals upon heat shock). 4) List of genes that are upregulated by tunicamycin treatment[1] and upon heat shock in an HSF-1 dependent manner[2]. Fold change induction of genes in wild type and in *hsf-1*(RNAi) background are also shown based on the results of Brunquell et al[2]. 5) List of genes that are upregulated by tunicamycin treatment[1] and upon heat shock in an HSF-1 dependent manner[3]. Fold change induction of genes in wild type and in *hsf-1*(sy441) background are also shown based on the results of Kovács et al[3]. 6) List of genes with promoters bound by HSF-1, as identified in the GTRD database[4]. 7) List of genes that are upregulated by tunicamycin treatment[1], and are also bound by HSF-1 based on the Gene Transcription Regulation Database[4]. 8) Putative HSEs found in the promoters of UPR^{ER} genes in *Caenorhabditis* species, using FIMO.

Table S2: Source data underlying Fig. 1 d, f; Fig 2 b, d; Fig. S1 d, f; Fig. S2 b-d is shown, which contains the statistical analysis of GFP intensities.

Table S3: Source data underlying Fig. 2 e, f; Fig. S3 a, b; Fig. S4 is shown, containing statistics for tunicamycin resistance assay

Table S4: Source data underlying Fig. 3 d, e; Fig. S6 a, -c is shown, which contains the statistical analysis for qPCR measurements

Table S5: Source data underlying Fig. 3 d, f; Fig. S6 a-c

The table contains the following data on tabs: 1) List of human genes that are upregulated by HSF1 and whose promoters are bound by HSF1, based on HSF1Base[5]. 2) List of genes that are upregulated by exposure to tunicamycin in human cultured primary fibroblasts[6]. 3) List of genes that are upregulated by exposure to tunicamycin in SV40-immortalized NS-SV-AC human salivary gland acinar cell line[7]. 4) List of genes that are induced by tunicamycin treatment[5] as well as upregulated and bound by HSF-1 according to HSF1base[5]. 5) List of genes induced by tunicamycin treatment[6] and identified as HSF-1bound and upregulated genes according to

HSF1base[5]. 6) Genes induced by both tunicamycin treatment[5,6] and identified as HSF-1 bound and upregulated targets according to HSF1base[5]. Brief functional descriptions are provided for each gene. 7) Overrepresented gene ontologies (Biological process) of genes that are induced by both, HSF1 and TM[7]. 5) List of HSEs identified in the promoter region of genes induced by tunicamycin treatment[5,6] and identified as HSF-1bound and upregulated genes according to HSF1base[5].

Supplementary Methods

Source of the analyzed transcriptomic data

No new transcriptomic data were generated during the project. We analyzed published transcriptomic data. Note that we did not reanalyze the raw data deposited in the Gene Expression Omnibus (GEO) database; rather, we used the gene sets provided by the authors in their publications or extracted them from the processed data files deposited in the GEO database.

1. Brunquell et al., 2016[2]: DEGs are available in the article, raw data deposited in NCBI SRA database (Access ID: SRP078295).
2. Kovács et al., 2024[3]: DEGs are available in the article, raw data deposited in NCBI GEO database with Accession number GSE241011.
3. Shen et al., 2005[1]: provides the processed microarray data as supplementary material in 'Table S3'.
4. Kovács et al, 2019[5]: processed data is available in the article and at https://hsf1base.org/.
5. Kelley et al, 2025[6]: DEGs were extracted from file GSE241261_normalized_counts_with_symbols.csv.gz, deposited along with the raw data at GEO under series GSE241261.
6. Yoon et al., 2023[7]: DEGs were extracted from file GSE226549_results_TN.csv.gz deposited along with the raw data available in the GEO database under the accession number GSE226549.

Supplementary References

1. Shen, X., Ellis, R. E., Sakaki, K. & Kaufman, R. J. Genetic Interactions Due to Constitutive and Inducible Gene Regulation Mediated by the Unfolded Protein Response in C. elegans. *PLOS Genetics* **1**, e37 (2005).
2. Brunquell, J., Morris, S., Lu, Y., Cheng, F. & Westerheide, S. D. The genome-wide role of HSF-1 in the regulation of gene expression in Caenorhabditis elegans. *BMC Genomics* **17**, 559 (2016).
3. Kovács, D. *et al.* Age-dependent heat shock hormesis to HSF-1 deficiency suggests a compensatory mechanism mediated by the unfolded protein response and innate immunity in young Caenorhabditis elegans. *Aging Cell* **23**, e14246 (2024).
4. Yevshin, I., Sharipov, R., Kolmykov, S., Kondrakhin, Y. & Kolpakov, F. GTRD: a database on gene transcription regulation—2019 update. *Nucleic Acids Research* **47**, D100–D105 (2019).
5. Kovács, D. *et al.* HSF1Base: a comprehensive database of HSF1 (heat shock factor 1) target genes. *International Journal of Molecular Sciences* **20**, 5815 (2019).
6. Kelley, L. P. *et al.* Integrated analysis of transcriptional and metabolic responses to mitochondrial stress. *Cell Reports Methods* **5**, (2025).
7. Yoon, J. *et al.* Resveratrol Attenuates the Mitochondrial RNA-Mediated Cellular Response to Immunogenic Stress. *International Journal of Molecular Sciences* **24**, 7403 (2023).
